# Cryptic splicing in ALS: from driving disease progression to unlocking novel therapeutics

**DOI:** 10.1146/annurev-genom-022024-011307

**Published:** 2026-04-21

**Authors:** Sara Emad El-Agamy, Francesca Mattedi, Pietro Fratta

**Affiliations:** 1 Department of Neuromuscular Diseases, UCL Queen Square Institute of Neurology, University College London, UK; 2 The Francis Crick Institute, London, UK

**Keywords:** cryptic splicing, axonal homeostasis, proteostasis, splice-switching therapeutics, gene therapy

## Abstract

TDP-43 is an RNA-binding protein that regulates multiple aspects of RNA processing, and its mislocalization from the nucleus to the cytoplasm is a defining feature of amyotrophic lateral sclerosis (ALS). While both loss and gain of function mechanisms contribute to disease, the discovery of cryptic splicing has shed light on the downstream consequences of TDP-43 nuclear clearance for neuronal health. Here, we highlight how loss of nuclear TDP-43 can drive a cascade of events leading to the impairment of cellular proteostasis and resulting in a positive feedback loop that perpetuates neuronal dysfunction. This sustains the appearance of cryptic splicing events in genes that are involved in key pathways for the maintenance of axonal homeostasis and synaptic transmission. In contrast to their detrimental effects on neuronal health, cryptic splicing mechanisms may be harnessed to develop novel therapeutic strategies, unprecedentedly expanding the availability of therapeutic avenues for TDP-43 proteinopathies.

## TDP-43 structure

TDP-43 is encoded by the *TARDBP* gene, which is located on human chromosome 1, and was originally identified as a suppressor of HIV-1 gene expression. TDP-43 is a ubiquitously expressed and highly conserved RNA/DNA binding protein, belonging to the heterogeneous nuclear ribonucleoprotein (hnRNP) family (26, 92, 123).

Full-length TDP-43 is 414 amino acids in length, with several functional domains. The N-terminal domain mediates TDP-43 homodimerisation to enhance its RNA processing activity (74, 155) and includes two RNA recognition domains (RRM), the nuclear localization signal (NLS) and a nuclear export signal (NES). The RRMs mediate TDP-43 binding to RNA and DNA through the preferential recognition of UG/TG-rich sequences, while the NLS facilitates the import of TDP-43 into the nucleus where it carries out its roles in transcription regulation and RNA processing (123). Disruption of the NLS leads to increased TDP-43 cytoplasmic levels and promotes its aggregation (160). Although both NLS and NES have been reported to affect the shuttling of TDP-43 between the nucleus and cytoplasm (160), the latter was shown to be dispensable as TDP-43 nuclear export occurs also by passive diffusion and independently of the export receptor CRM1/Exportin-1 (46, 120). TDP-43 also harbors three motifs that mediate its import into mitochondria (156). The C-terminal domain (CTD), also known as the low complexity domain (LCD), is characterized by a glutamine/asparagine-rich and a glycine-rich region. It has been reported to regulate TDP-43 solubility and multiple studies support its prion-like and aggregation prone properties (123).

## TDP-43 localization and functions

In the nucleus, TDP-43 regulates gene expression at different levels. Indeed, it can bind ssDNA TG repeats and is found at promoter and enhancer regions where it regulates the transcription of several targets, while multiple studies report its involvement also in chromatin remodeling (58). In addition to this, TDP-43 localizes at sites of DNA damage, where it participates in the DNA double-strand break repair pathway through non-homologous end joining (58).

TDP-43 has a pivotal role in RNA processing and splicing. Originally, it was shown to regulate the splicing of *CFTR* mRNA through binding to UG-rich regions (26). CLIP-seq technology then allowed the wider characterization of the transcripts regulated by TDP-43, revealing it binds to over 6000 targets. Although TDP-43 binding sites were found also in long non-coding RNAs and in 3’UTRs, the majority lie within intronic regions (122, 145). TDP-43 acts as a splicing repressor, but can also function as a splicing enhancer, and can regulate polyadenylation and miRNA processing (27, 79, 170).

Although TDP-43 resides mainly in the nucleus, several cytoplasmic functions have also been reported (16). TDP-43 binds the 3’UTR of several transcripts, regulating their stability and translation (16, 121). The regulation of transcripts localization, stabilization and translation is particularly important in neurons, where mRNAs assembled in ribonucleoparticles (RNPs) are transported along axons to be locally translated at distal sites. TDP-43 is found in RNPs, supporting its role in mRNA transport (3, 62). TDP-43 is also recruited to stress granules (SGs), and modulates their dynamics by regulating G3BP1 (Ras GTPase-activating protein-binding protein 1) level (9, 105). Moreover, mitochondrial dysfunction is a prevalent feature in ALS and, although wild-type TDP-43 can also localize to mitochondria, ALS-causing mutants abnormally accumulate in these organelles potentially driving toxicity (16, 156).

## TDP-43 proteinopathies

Ubiquitinated cytoplasmic inclusions had long been described in amyotrophic lateral sclerosis (ALS) and frontotemporal dementia (FTD) postmortem samples and in 2006, TDP-43 was identified to be the main ubiquitinated protein (19, 138). The cytoplasmic aggregates contain 25-35 kDa C-terminal fragments that are N-terminally-truncated and hyperphosphorylated TDP-43 (138). TDP-43 cytoplasmic aggregation is not spatially limited to the somatic compartment as evident by the accumulations detected within intramuscular axonal bundles of ALS patients (89, 125) and in neurites in a subset of TDP-FTD (type C) (142). Crucially, TDP-43 is also depleted from nuclei, where it is mainly localized in physiological conditions.

In 2008, ALS-causative mutations were identified in *TARDBP*, clustering in the CTD but also spanning other protein domains (77, 136). Although these mutations account for only 0.5% of all ALS cases, their identification was crucial in supporting a pathogenetic role for TDP-43 in disease. Subsequently, TDP-43 pathology was reported in many disorders including Alzheimer’s disease (AD), limbic-predominant age-related TDP-43 encephalopathy (LATE), Perry syndrome and inclusion body myositis (IBM), sparking extensive research into its role in neuropathogenesis (19). Now, ‘TDP-43 proteinopathies’ refer to a spectrum of these progressive neurodegenerative diseases pathologically characterized by TDP-43 nuclear depletion and cytoplasmic aggregation. Whilst in AD TDP-43 pathology co-occurs in a subset of cases along with the established Tau and Amyloid-β pathology, all ALS cases, with the exception of ~3% caused by *SOD1* and *FUS* mutations, and ~45% of FTD cases (FTD-TDP) are primarily characterized by TDP-43 pathology.

ALS results in the selective demise of upper and lower motor neurons leading to progressive muscle weakness and death within 2-5 years from the onset of symptoms due to respiratory failure (104). Cortical neurons are predominantly impacted in FTD culminating in cognitive deficits and behavioral alterations (104). ALS and FTD were initially viewed as distinct entities. However, a growing body of evidence highlighting clinical overlap, mirrored by genetic and pathological similarities, supports viewing these disorders as a continuum comprising the ALS-FTD spectrum (95, 104). While about 50% of FTD patients have a familial history of the disease, the majority of ALS cases are sporadic (~90%) (95).

Currently, there is no approved cure or disease-modifying therapy for sporadic ALS or FTD, other than riluzole which has a small effect on survival, and Tofersen, which is only approved for SOD1-ALS cases. Thus, delineating the early changes triggering neuronal dysfunction is crucial for the identification of potential therapeutic targets.

## TDP-43 loss and gain of function

In TDP-43 proteinopathies, TDP-43 mislocalizes from the nucleus to the cytoplasm, where it aggregates. This has led to both loss and toxic gain of function (LOF, GOF) mechanisms proposed as drivers of neurodegeneration. Notably, conditional depletion of TDP-43 in spinal motor neurons in mice is sufficient to induce a progressive motor phenotype reminiscent of that observed in human ALS cases (44, 162). Conversely, neuromuscular phenotypes are found without TDP-43 LOF in mouse models with endogenous *Tardbp* mutations and in mice overexpressing TDP-43, overall showing that both TDP-43 LOF and GOF are likely to contribute to neurodegeneration (138).

Recent reports show that seeding of TDP-43 LCD fibrils or patient derived aggregates can drive TDP-43 LOF and illustrate how cytoplasmic misfolding and nuclear loss intertwine in disease pathogenesis (101, 128). Crucial outstanding questions now include ‘What are the upstream triggers leading to aggregation and nuclear depletion? What is the temporal relationship between the two events?’. Axonal damage and trauma can induce TDP-43 mislocalization, and intriguingly, traumatic brain injury is an established risk factor for neurodegenerative diseases (18, 54, 65). How aging and other environmental and genetic factors contribute to disease onset remains largely unknown.

In this review, we will discuss the molecular mechanisms that position TDP-43 loss of function as an initiating and self-propagating driver of neurodegeneration in ALS and FTD, with a particular focus on RNA processing deficits and their downstream consequences on cellular proteostasis, axonal homeostasis and synaptic transmission.

## Loss of TDP-43 nuclear function: cryptic splicing and other post-transcriptional events

Because of TDP-43's crucial role in RNA processing, a lot of attention has recently been dedicated to the consequences of its nuclear LOF. RNA-seq data from TDP-43 depleted neurons show widespread transcriptomic alterations. In particular, the Wong group described the inclusion of intronic regions that are normally repressed, referred to as cryptic exons (CEs), into mature transcripts (2, 94, 106). These events can show strong specificity *in vitro*, sometimes being never detected in physiological conditions throughout all publicly available data, whilst are specifically detected in postmortem tissues of patients affected by ALS and FTD, and also in AD and IBM (23, 60, 67, 94, 99, 124, 141). Whilst in a number of cases CEs are a cassette exon emerging from an intron, cryptic splicing and generally cryptic RNA processing can be broader. In some cases, only a 5’ or 3’ splicing junction is cryptic, leading to the change of an existing exon, or a novel exon skipping event (skiptic exon) can emerge, leading to a shorter transcript. In other instances, lack of TDP-43 leads to novel polyadenylation events: a) a novel exon definition can induce premature polyadenylation leading to a truncated transcript, as is the case with *STMN2* (108); b) polyadenylation can be induced within an unspliced intron (intronic polyadenylation, IPA) also leading to a truncated transcript, or c) novel polyadenylation sites can be used within the 3’UTR leading to either 3’UTR extension or contraction (6, 25, 171).

**Figure 1:**
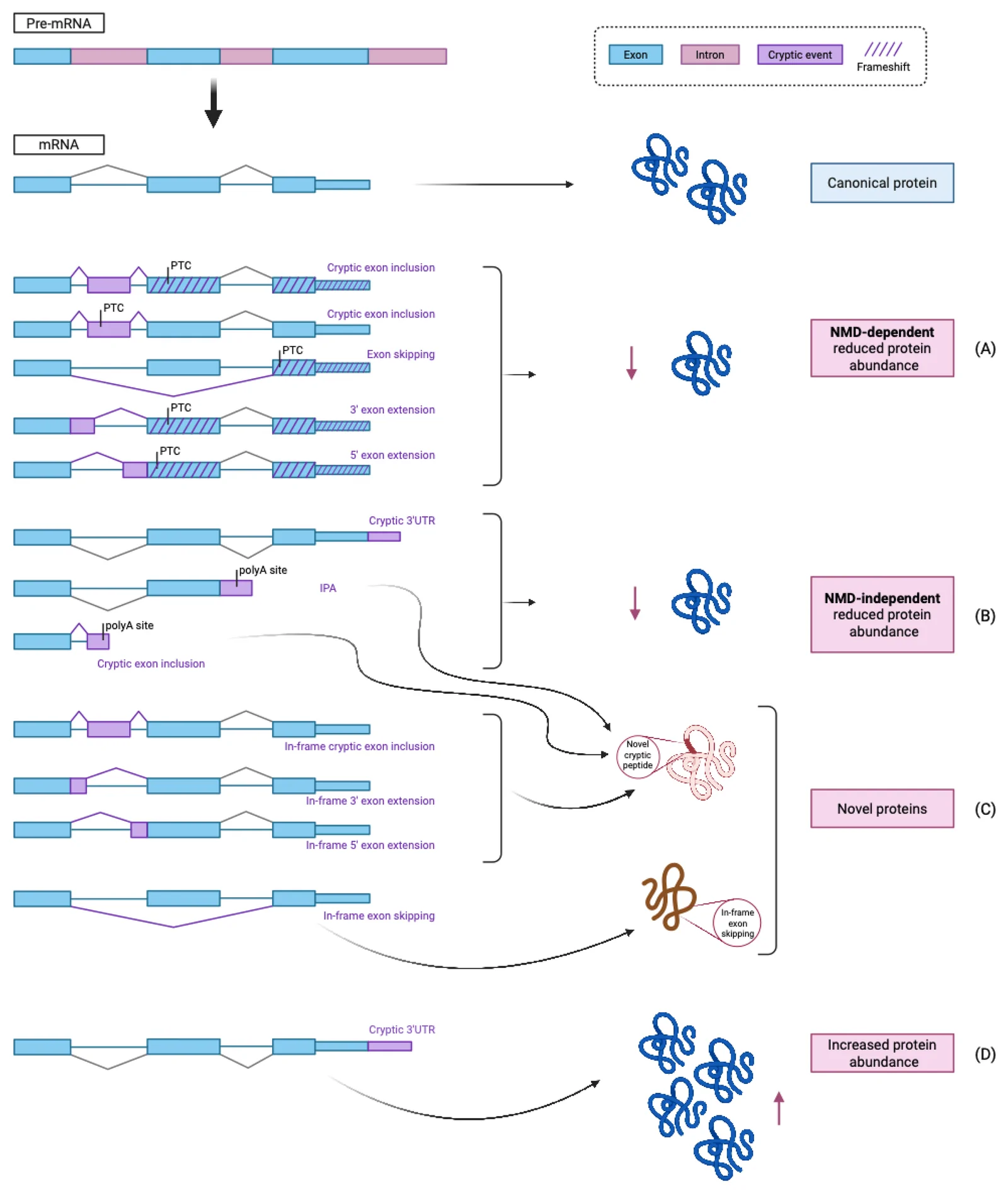
The effects of cryptic splicing on the cellular proteome. A) Cryptic and skiptic events can lead to a loss of protein by introducing a premature termination codon (PTC), which results in the transcript being degraded through NMD. B) A reduction of protein levels can occur independently from NMD through the inclusion of an early polyadenylation site, leading to the production of a truncated protein, or changes in the 3’ UTR. C) When cryptic and skiptic splicing events occur in-frame, they lead to the production of novel proteins. In the former case, cryptic peptides are produced. D) Cryptic events in the 3’UTR can lead to an increase in protein abundance by altering the transcript stability and translation efficiency. (Figure created with BioRender: https://biorender.com/kcu86cx)

CEs can have different consequences for the encoded proteins leading to protein loss, the formation of a different protein or an increase in protein levels (Figure 1). Several CEs include stop codons or cause a frameshift resulting in the insertion of a premature termination codon, leading to transcript degradation through the non-sense mediated decay (NMD) pathway ultimately resulting in a reduction of the protein levels (24, 133, 172). This is the case for the CEs included in *UNC13A* (23, 99) and *ATG4B* (146, 147). A similar mechanism can be triggered by exon skipping events inducing an out of frame change. Protein loss can also be the consequence of premature polyadenylation, especially when this occurs at the very beginning of the open reading frame, as is the case with *STMN2* (Figure 1B) (82, 108).

When cryptic cassette exon, 5’ splice site (SS), 3’SS and skiptic events are in-frame and do not contain stop codons, this leads to the formation of a novel protein (Figure 1C). When a novel protein sequence is included, it is referred to as cryptic peptide (69, 129). Cryptic peptides are also predicted to be produced when premature polyadenylation or IPAs occur in introns lying between coding exons. Several cryptic peptides have been detected in the cerebrospinal fluid of ALS patients, such as the one inserted into the HDGFL2 protein (30, 69, 129). The inclusion of a cryptic peptide in HDGFL2 has also been shown to change the protein’s binding partners and possibly its function (129). Similarly, the alteration of protein sequence induced by a skiptic exon in *KCNQ2* leads to a dominant negative effect (76). Recently, cryptic peptides have been identified as T cell antigens triggering immune response (35), which highlights their relevance in ALS pathobiology.

Finally, whilst cryptic 3’UTR events do not impact the coding sequence, they can impact RNA stability and lead to an increase in protein levels (Figure 1D), as shown for *ELK1* and *MARK3* (6, 25), or can contribute to a reduction in protein levels (Figure 1B), as reported for *NEFL* (171).

Taken together, such aberrant splicing affects a variety of pathways in the cell. Interestingly, while some events are ubiquitous, others vary depending on cell type (73). As many of them affect key genes for neuronal function, it is conceivable that they could synergistically contribute to the degeneration of neuronal cells. Here, we review how key mechanisms of neuronal biology are affected by TDP-43 nuclear LOF. In particular, we discuss how TDP-43-dependent defects in pre-mRNA splicing contribute, at least in part, to altered cellular proteostasis, axonal homeostasis and neuronal activity in ALS and FTD.

## Positive feedback loop: Merging disruption of RNA and protein regulation

A common feature of TDP-43 proteinopathies is the progressive disease trajectory. Seeding of TDP-43 LCD fibrils or patient derived aggregates has been reported to drive the nuclear clearance of TDP-43 (101, 128). Below, we highlight how TDP-43 LOF can exacerbate cytoplasmic accumulations, instigating a toxic positive feedback loop that drives pathogenic progression.

### TDP-43 autoregulation

TDP-43 protein levels are tightly controlled through an autoregulation mechanism involving TDP-43 binding to its own 3’UTR, influencing splicing, alternative polyadenylation signal (PolyA) selection, and nuclear retention (10, 87). TDP-43 is mostly translated from a transcript in which exons 1-6 are included and the PolyA1 within the 3'UTR is selected. TDP-43 can bind and promote the splicing of intron 7, resulting in loss of PolyA1 and formation of a highly unstable transcript that is retained in the nucleus and likely cleared through exosomal-mediated degradation (10, 87). TDP-43 binding can also promote the splicing of introns 6 and 7, producing a *TARDBP* variant that lacks the intrinsically disordered region (*ΔIDR-TARDBP*), and harbors a premature termination codon, culminating in its degradation via NMD. However, a pool of *ΔIDR-TARDBP* can escape NMD generating a truncated TDP-43 protein (165). This ΔIDR-TDP-43 protein can heterodimerize with cytoplasmic TDP-43 and recruits HSPA8 (Heat Shock Protein Family A (Hsp70) Member 8), so producing a heterocomplex that is targeted for degradation via chaperon-mediated autophagy (165).

A 3’UTR missense variant within *TARDBP* intron 7 was reported in ALS-FTD postmortem samples, highlighting the significance of the autoregulation process in TDP-43 proteinopathy (59). An age-dependent decline of *TARDBP* alternative splicing was reported in brains of aged healthy subjects, aligning with aging being a primary risk factor for ALS-FTD (165). The gradual nuclear clearance observed during disease promotes the utilization of *PolyA1 TARDBP*, increasing the overall mRNA and protein abundance and likely enhancing the cytoplasmic aggregation (87). Indeed, frontal cortices of TDP-43 proteinopathy cases showed correlation between impaired *TARDBP* splicing and *STMN2* expression, a sensitive marker of TDP-43 LOF (165).

**Figure 2:**
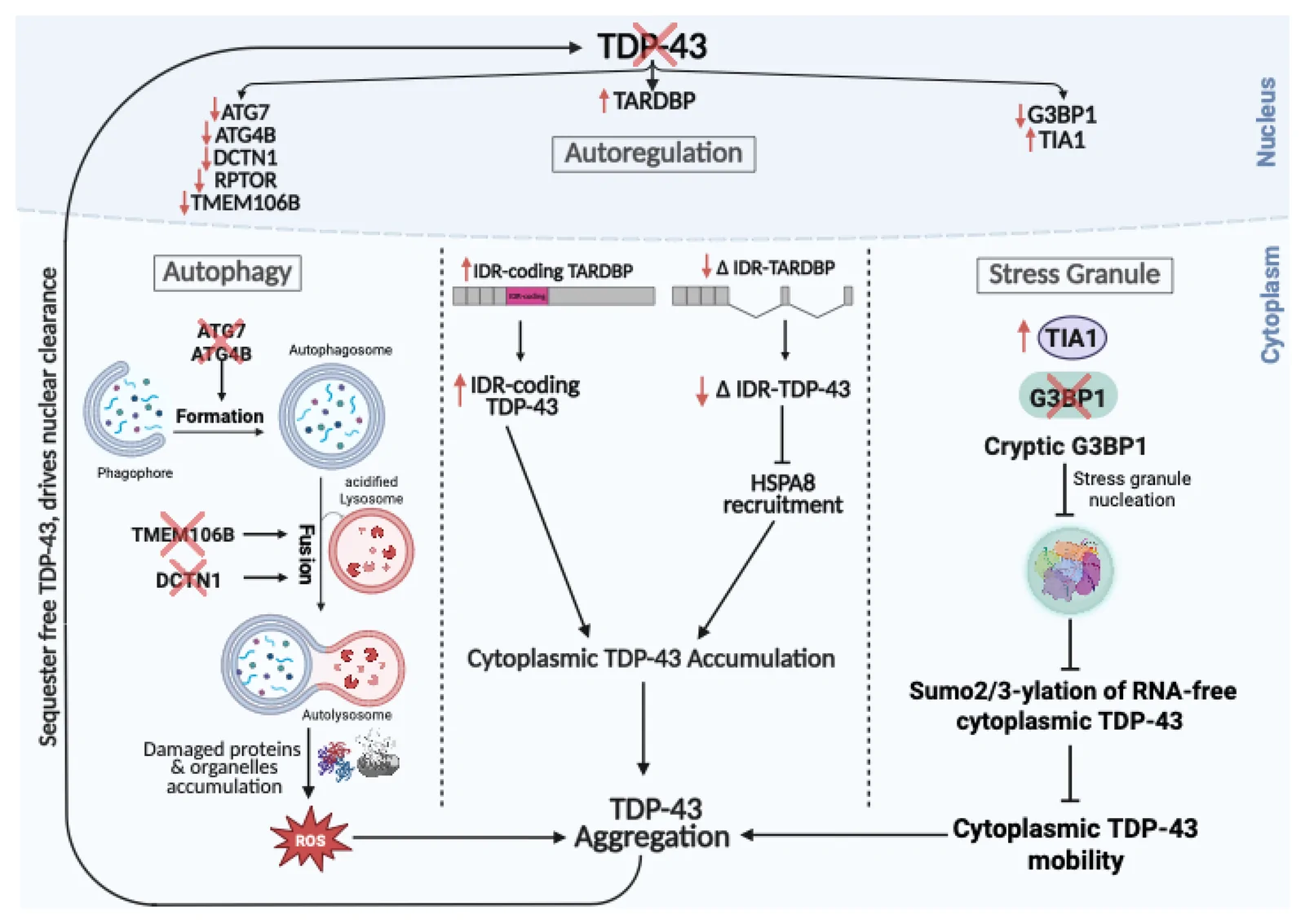
A positive feedback loop linking nuclear depletion and cytoplasmic aggregation. Loss of nuclear TDP-43 promotes its cytoplasmic aggregation by disrupting autophagic degradation, autoregulation, and stress granule dynamics. Impaired autoregulation increases cytoplasmic *TARDBP* level and limits chaperone-mediated clearance of TDP-43 (middle panel). In parallel, defective autophagic degradation leads to the accumulation of damaged proteins and organelles, instigating oxidative stress (left panel). The impaired SG assembly further reduces the mobility of RNA-free cytoplasmic TDP-43, facilitating its aggregation (right panel). Together, increased cytoplasmic TDP-43, oxidative stress and reduced mobility drive irreversible aggregation, which sequesters functional TDP-43, exacerbating nuclear depletion and establishing a self-sustaining positive feedback loop that drives neurodegeneration. (Figure created with BioRender: https://biorender.com/zv3cip3)

ASOs blocking the binding sites of hnRNPA1 and hnRNPC, two key repressors of *TARDBP* alternative splicing, restore *TARDBP* splicing and mitigate disease progression in *in-vitro* and *in-vivo* TDP-43 proteinopathy models (165). In contrast, bypassing autoregulation and overexpression of ΔIDR-TDP-43 induces cellular toxicity through excessive sequestration of functional TDP-43 (45). Thus, autoregulation is an intricate mechanism to safeguard TDP-43 level both at the RNA and protein level. Recent reports have shown that seeding TDP-43 LCD fibrils or patient derived aggregates can recapitulate key disease signatures and impair autoregulation in cellular models (101, 128). These findings offer a mechanistic link between loss and gain of function paradigms and highlight how disrupted autoregulation feeds a toxic forward loop that exacerbates disease (Figure 2, middle panel). While several therapeutic strategies target downstream consequences of TDP-43 LOF, rescuing *TARDBP* autoregulation could control the pathological positive feedback loop.

Although TDP-43 LOF is clearly implicated in driving neurodegeneration, efforts to restore TDP-43 level must be approached with caution, as increased nuclear TDP-43 can lead to GOF toxicities with distinct splicing signatures. Increased TDP-43 levels lead to the skipping of exons that are otherwise constitutively included in mature transcripts under physiological TDP-43 level (5, 31, 52, 157).

### TDP-43 and protein clearance pathways

The ubiquitin-proteasomal and the autophagy-lysosomal systems are the two main pathways responsible for protein degradation. They mediate the clearing of soluble and aggregated TDP-43 respectively. Thus, it is not surprising that mutation of autophagy-related genes including *sequestosome-1*/P62, *optineurin, valosin-containing protein* (*VCP*) and *ubiquilin-2* are associated with ALS-FTD (95). Interestingly, TDP-43, an autophagy substrate, regulates different stages of autophagy through multiple RNA processing events. This results in another mechanism for regulating its cellular abundance as well as global protein and organelle clearance.

#### ATG7

TDP-43 LOF directly impairs autophagy through downregulating autophagy related genes (ATG) that are involved in autophagosome formation. TDP-43 binding to *ATG7* is necessary for its stabilization, the mechanism of which remains to be elucidated (22, 164). The contribution of ATG7 to cellular homeostasis is highlighted by *Atg7* null mice that die within a day of birth (85). CNS-specific depletion of *Atg7* resulted in behavioral and motor deficits due to massive neuronal loss (84). Importantly, inclusion bodies of polyubiquitinated proteins were detected in Atg7 deficient neurons and their accumulation worsened with age, offering a direct link between autophagy and neurodegeneration (84). Results from *Atg7* deficient mouse models were corroborated through *ATG7* association with neurological disorders in humans (36). Twelve patients from five families with recessive *ATG7* mutations had intellectual and motor deficits, a result of neuro- and myopathic autophagy deficits (36). In adulthood, marked reduction of ATG7 has been reported in ALS-FTD postmortem samples exhibiting TDP-43 inclusions and nuclear clearance (43).

#### ATG4B

Besides ATG7 loss, TDP-43 nuclear clearance has been implicated in cryptic splicing of *ATG4B* (146). Here, the CE inclusion introduces a premature stop codon that marks the transcript for degradation via NMD. The pool of cryptic transcripts that manage to evade degradation results in a truncated protein that lacks the phosphorylation domain promoting ATG4B hydrolase activity (146). While *Atg4b* null mice develop normally and display only mild autophagic deficits, *Atg4b* depletion in *SOD1*^G93A^ mutant mice dramatically reduces their lifespan, highlighting its necessity under autophagic stress conditions (147). *ATG4B* CE inclusion correlates negatively with disease duration in ALS suggesting pathological contribution to the disease (146). Although an ASO targeting *ATG4B* was able to rescue its cryptic splicing, its ability to rescue autophagy function and efficacy in preclinical models remain to be evaluated (147).

Besides autophagic deficits, ATG4B mutation negatively impacts microtubule dynamics through impairing LC3 (MT-associated protein 1 light chain 3) lipidation, culminating in cargo accumulations and axonal swellings (114, 146). Axons mostly rely on the biosynthetic and degradative capacity of the cell body to maintain themselves, thus bidirectional microtubule-dependent transport of organelles, proteins, RNAs and other molecular complexes is crucial for neuronal survival and homeostasis (100). Indeed, autophagy is closely linked to axonal transport. Autophagosomes are constitutively generated at axonal tips and some degradation can occur locally through continuous delivery of degradative lysosomes (50), however, the main pool of enzymatically active lysosomes reside in the soma which necessitate efficient dynein-dependent shuttling (32).

#### TFEB and DCTN1

TDP-43 LOF leads to the degradation of raptor, a positive regulator of mTORC1 (mammalian target of rapamycin complex 1) activity, resulting in increased nuclear translocation of TFEB (transcription factor EB) and upregulation of autophagic and lysosomal biogenesis genes (164). While this upregulation seems beneficial at first glance, it is accompanied by an impairment of autophagosomes-lysosomes fusion due to destabilization of *DCTN1* (dynactin 1), the main subunit of dynactin (164). Dynactin is an indispensable co-factor for initiating dynein-mediated retrograde transport (132). Dynactin mutations have been linked to ALS and Perry syndrome, two neurodegenerative diseases characterized by TDP-43 inclusions (86). Mutations of tubulin, motor and adaptor proteins (*TUBA4A, KIF5, ANXA11*) are also associated with ALS (119), highlighting the significance of axonal transport in autophagic clearance and overall neuronal homeostasis. Taken together, increased TFEB nuclear localization along with DCTN1 depletion impairs autophagic flux and results in the accumulation of immature autophagic vesicles, triggering neurotoxicity (164).

#### TMEM106B

TMEM106B, a transmembrane protein embedded into late endosomes and lysosomes, is an established risk factor for FTD and LATE (174). TMEM106B regulates multiple aspects of the endolysosomal degradative capacity. In addition to promoting acidification through vacuolar ATPase activity, TMEM106B regulates the retrograde trafficking of endolysosomal vesicles (174). Lengthening of the 3’UTR of *TMEM106B* has been reported in cellular TDP-43 LOF models as well as FTD postmortem samples (171). The 3’UTR extension culminated in a reduction in TMEM106B dimer levels, albeit the mechanism driving dimer but not monomer down regulation remains to be investigated (171). Loss of TMEM106B worsens *C9orf72* dipeptide repeat pathology by disrupting autophagosome maturation (11). Moreover, TMEM106B depletion leads to the accumulation of enlarged endolysosomes at the axon initial segment (98, 171).

### TDP-43 LOF and stress granules dynamics

Stress granules (SGs) are dynamic membrane-less condensates that assemble in response to a variety of cellular stressors (102). SGs formation is concurrent with a translational arrest through sequestration of stalled transcripts at the translation initiation phase. SGs mediate a protective stress response by acting as a site for mRNA triage, in which transcripts are selectively sorted and routed for re-initiation of translation, degradation, or storage (131). Beyond RNA, SGs sequester misfolded proteins and apoptosis regulatory factors and enzymes to preserve the cellular degradation capacity and prevent the activation of apoptotic pathways (38). Interestingly, SGs also stabilize ruptured endolysosomal membrane allowing time for repair (29). While the proteomic composition of these granules varies according to the cell type and the stress-inducing context (102), G3BP1 acts as the central node for nucleation of SGs (131).

#### G3BP1 and TIA1

TDP-43 supports SGs assembly through modulating two key regulators of SGs dynamics, G3BP1 and TIA1 (T cell intracellular antigen-1) (105). TDP-43 regulates the stability and splicing of *G3BP1* through binding to the 3’UTR and intronic regions respectively. TDP-43 depletion destabilizes the short, most abundant *G3BP1* transcript (131), and introduces a neuronal-specific in-frame CE, resulting in a dominant negative G3BP1 isoform that hampers SG nucleation (49). TDP-43 loss also delays the assembly of TIA-1 granules, an LCD-containing RNA-binding protein (RBP) that promotes SG assembly, despite increasing the overall TIA-1 mRNA and protein level (105). Under oxidative stress conditions, SGs serve an additional function in regulating the solubility/mobility of the cytoplasmic RNA-free pool of TDP-43 by acting as a preferential site of Sumo2/3-ylation (153) (Figure 2, right panel). TDP-43 acetylation, another post-translational modification that promotes cytoplasmic TDP-43 aggregation, occurs on the same residues as sumoylation. Thus, TDP-43 recruitment to SGs could be a quality control mechanism for aggregation-prone RBPs including TDP-43 (153). Interestingly, knockdown of *DCTN1* in *Drosophila*, mirroring a TDP-43 LOF event, delayed SGs disassembly during stress recovery leading to the formation ubiquitin-positive TDP-43 aggregates (149). As mentioned previously, dynactin mutations have been linked to ALS and Perry syndrome.

Since SGs concentrate aggregation-prone RBPs, they are suspected to be a culprit that facilitates protein aggregation. However, whether TDP-43 recruitment to SGs could instigate its aggregation is debatable. Several lines of evidence have shown that stress-induced cytoplasmic TDP-43 oligomerization or aggregation is spatially independent of SGs (56, 137). However, work from the Hyman lab has recently shown that increasing TDP-43 concentration beyond a certain threshold, combined with oxidative stress, results in intra-condensate demixing of TDP-43 inside SGs with liquid-to-solid transition leading to the formation of pathological aggregates (166). Beyond seeding TDP-43 aggregates, SGs also sequester HDAC6 (histone deacetylase 6), a proteostasis factor that regulates autophagy-mediated clearance of various proteins including TDP-43, hence enhancing its aggregation (34). Collectively, these findings suggest the involvement of SGs in concentration-dependent liquid-to-solid transition of TDP-43 condensates. These persistent aggregates exhibit the pathological features of ubiquitination and phosphorylation (166) and could further exacerbate TDP-43 LOF by sequestering free TDP-43 along with other vital molecules.

### Impact on other RNA Binding Proteins

TDP-43 loss leads to cryptic events in transcripts encoding other RBPs, beyond G3BP1 and TIA-1. The dysregulation of these proteins can lead to multiple downstream RNA changes. ELAVL3 is an RBP with enriched expression in neurons that is strongly dysregulated in ALS (41). The inclusion of a CE has been detected between exon 3 and 4 of *ELAVL3* mRNA in TDP-43 depleted iPSC-derived neurons and in postmortem tissues from ALS patients, leading to a decrease of ELAVL3 levels (37, 41). ELAVL3 abnormalities in spinal neurons were detected more frequently than TDP-43 mislocalization and appeared earlier in postmortem spinal neurons, indicating that other factors are likely to be involved in its regulation (41). *Elavl3* knockout mice have seizures and motor phenotypes consistent with progressive cerebellar ataxia, likely through dysregulation of genes involved in glutamate synthesis and neurotransmission (68). Moreover, Purkinje neurons depleted of ELAVL3 displayed altered mitochondrial trafficking and axonal swellings filled with axonal cargoes and also somato-dendritic proteins such as MAP2 (116). These swellings could act as foci that promote further aggregation of TDP-43 along with other aggregation-prone proteins.

CELF5 is an RBP involved in alternative splicing and preferentially enriched in the nervous system (83, 172). TDP-43 has been shown to repress the inclusion of a CE that renders *CELF5* transcript NMD sensitive (172). CELF5 was the third most downregulated protein in iPSC-derived neurons depleted of TDP-43 (129, 172). Interestingly, *CELF5* emerged as one of the differentially expressed transcripts that distinguish vulnerable from resistant motor neurons, suggesting its potential involvement in motor neuron disease (83).

The wide-spread splicing alterations observed in TDP-43 pathology are not exclusively mediated through the nuclear LOF. Cytoplasmic TDP-43 sequesters several splicing regulators, promoting the missplicing of amyloid precursor protein (*APP*) and increasing the amyloid-β burden (175). *APP* missplicing has been shown in TDP-43 depleted neurons of ALS/FTLD-TDP postmortem brains (175).

Taken together, the nuclear depletion of TDP-43, which occurs early in the course of the disease, triggers a positive feedback loop of aberrant RNAs and impaired protein homeostasis that drives disease progression (Figure 2). The nature of the event(s) triggering the nuclear clearance of TDP-43 remains largely enigmatic despite extensive research over the last two decades. Defective autoregulation and autophagic processing are key effectors of TDP-43-dependent pathogenesis. Loss of nuclear TDP-43 disrupts its autoregulatory feedback loop, increasing the overall *TARDBP* levels (Figure 2, middle panel). In parallel, TDP-43 LOF limits the mobility of the cytoplasmic RNA-free pool of TDP-43 through impairing SGs dynamics (Figure 2, right panel). Besides augmenting TDP-43 cytoplasmic accumulations, autophagy impairment elicits damaged proteins and organelle accumulation which in turn produce cellular stress (90). The increased TDP-43 accumulation in the cytoplasm can promote TDP-43 concentration into SGs and along with oxidative stress, an established outcome of an overwhelmed autophagic pathway (Figure 2, left panel), leading to liquid-to-solid transition of TDP-43 and irreversible aggregation. These aggregates promote further nuclear loss of TDP-43 creating a self-sustaining positive feedback loop that contributes to pathology and neurodegeneration.

The mechanisms described above likely trigger a plethora of downstream effects on neuronal biology, synergistically contributing to neurodegeneration. In particular, mounting evidence highlights how ALS-affected neurons are characterized by defects in pathways involved in sustaining axonal homeostasis and synaptic transmission (33, 57, 121).

## How does TDP-43 nuclear LOF affect axonal functionality?

**Figure 3:**
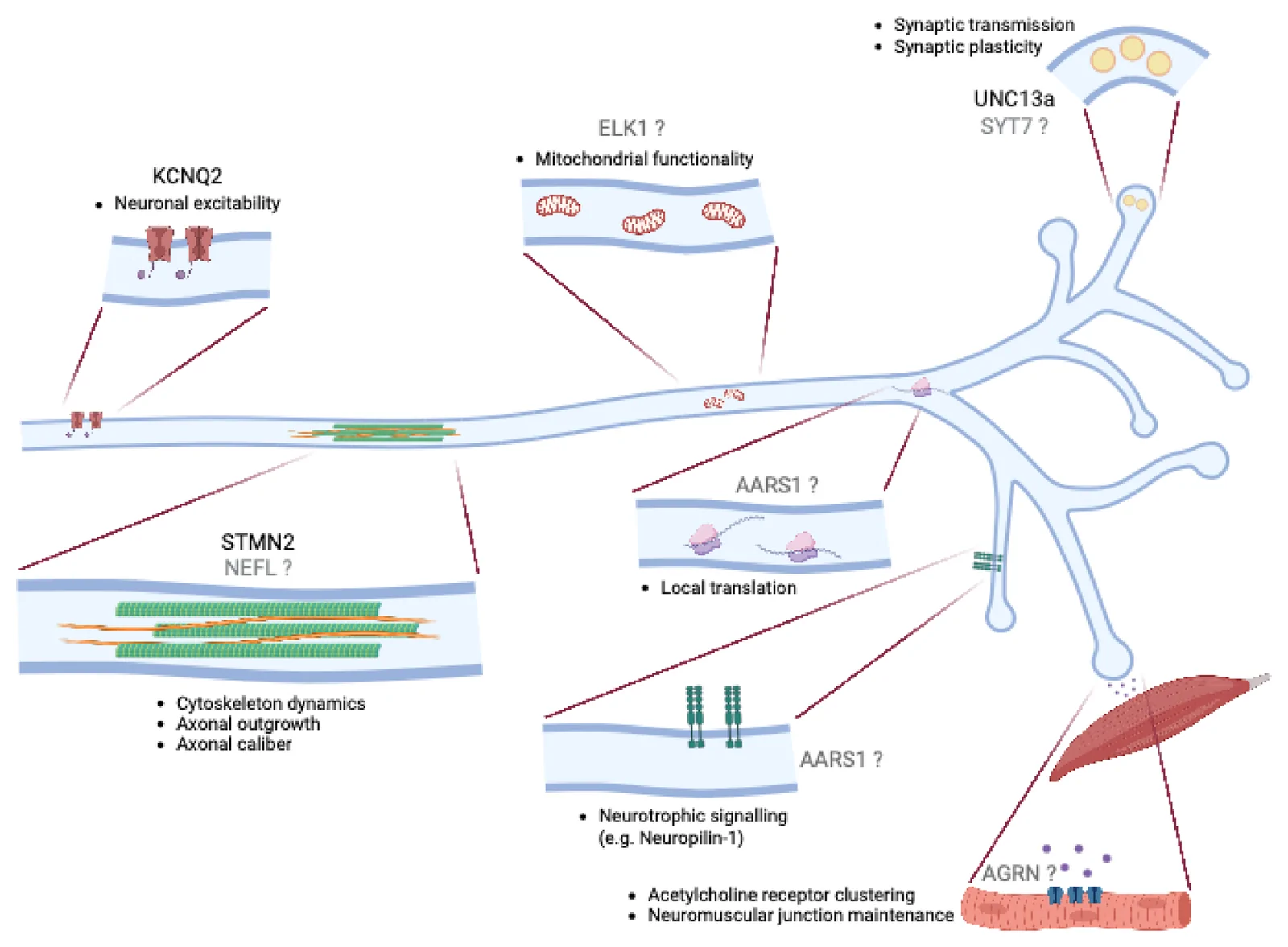
Axonal pathways affected by cryptic splicing. Cryptic splicing events have been detected in several genes involved in various processes crucial for the maintenance of axonal homeostasis. While evidence of their contribution to neuronal dysfunction has already been reported for some targets (in black) in the context of TDP-43 nuclear loss, other putative ones have emerged (in grey), opening interesting new lines of research to further characterize axonal dysfunction downstream TDP-43 LOF. (Figure created with BioRender: https://biorender.com/g8ne4in)

Neurons have a peculiar cellular architecture, with axons that can extend for long distances (100). Thus, they face the challenge of sustaining cellular processes that occur far from the soma, which conceivably makes neurons more sensitive to alterations in the expression of genes involved in the regulation of cytoskeleton dynamics, local protein synthesis, intracellular trafficking and ensuring the integrity of peripheral cellular structures such as neuromuscular junctions (NMJs). Remarkably, loss of TDP-43 leads to the inclusion of CE in transcripts involved in these pathways (Figure 3).

### Disruption of cytoskeleton dynamics in axons

#### STMN2

In 2019, loss of TDP-43 nuclear function was shown to affect the splicing of *STMN2*, leading to the inclusion of a CE and an early polyadenylation site. As a result, the levels of full-length *STMN2* are dramatically reduced (82, 108). The inclusion of *STMN2* CE has been observed in postmortem tissues of patients affected by ALS and also by TDP-43-associated FTD and AD, highlighting its importance as a potential biomarker (1, 82, 108, 124). Importantly, STMN2 plays a key role in neurite outgrowth and several studies have shown that its loss leads to axonal shrinkage, NMJ denervation and alteration of axonal transport in *Drosophila* and in mice, where it is sufficient to cause motor and sensory phenotypes (64, 88, 91, 97, 144). Moreover, loss of STMN2 impairs axonal regeneration after axotomy in cultured motor neurons (13, 82, 108) and after nerve crush in mice (13, 91). Although it’s now clear that STMN2 plays a crucial role in axonal maintenance and neurite outgrowth, the mechanism of action is not fully understood.

STMN2 is involved in the regulation of microtubule dynamics and has been reported to bind tubulin heterodimers (144). While STMN2 promotes microtubule catastrophe *in-vitro*, loss of the single *stathmin* gene in *Drosophila* was reported to cause microtubule destabilization (144), and a decrease in the levels of polymerized β3 tubulin was observed in mice and iPSC-derived neurons upon STMN2 loss (64). On the other hand, others have reported only mild defects in microtubule assembly in STMN2 depleted iPSC-derived neurons and linked motor axons collapse in *Stmn2* knockout mice to reduced neurofilament spacing rather than altered microtubule density (13, 97). Intriguingly, although STMN2 is thought to regulate microtubule dynamics in axons by contributing to maintain a balance between microtubule polymerization and depolymerization, its ability to promote axonal regeneration has recently been proposed to be independent from its role in microtubule dynamics, and membrane-bound STMN2 might instead be driving axon protection mechanisms, possibly by mediating the transport of cargoes essential for axonal maintenance (13, 40, 143).

Remarkably, restoring STMN2 levels rescues, at least in part, axonal regrowth defects in TDP-43 depleted neurons (12, 82, 108), as well as axonal outgrowth deficits in iPSC-derived neurons from spinal muscular atrophy (SMA) patients and neuromuscular phenotypes in an SMA mouse model (118). Although the rescue of STMN2 levels significantly improves the ability of axons to recover, it is important to consider that TDP-43 mislocalization affects other aspects of axonal functionality, such as synaptic transmission, NMJ integrity, cytoskeleton dynamics and axonal transport. Therefore, addressing the consequences of other cryptic splicing events is crucial to comprehensively understand the effects that TDP-43 loss has on axonal homeostasis.

#### NEFL

Loss of TDP-43 also results in an alternative polyadenylation event in the neurofilament light chain (*NEFL*) transcript, leading to an elongation of the 3’UTR which ultimately reduces NEFL protein levels (171). Interestingly, *NEFL* levels have been reported to be affected by TDP-43 loss also through the downregulation of two miRNAs (miR-b1336 and miR-b2403) involved in the stabilization of the transcript (70). It is likely that these two mechanisms jointly contribute to the decrease of NEFL protein observed in ALS. NEFL is a key component of the axonal cytoskeleton and in particular of neurofilaments, together with neurofilament heavy and medium chains (NEFH and NEFM), α-internexin and peripherin. Neurofilaments have various roles in axons, as they contribute to the maintenance of axonal architecture, regulate axonal caliber and radial growth, as well as organelles docking and microtubule assembly (20). Interestingly, both dominant and recessive mutations in NEFL cause axonal neuropathies, highlighting its role in axonal maintenance (75, 169). Moreover, *Nefl* knockout mice show reduced axonal caliber and amplitude of miniature excitatory postsynaptic current. Axonal regeneration is unaffected by NEFL loss, while mitochondrial transport is increased, possibly interfering with the regulation of mitochondrial positioning along the axons in response to cellular needs (127). Taken together, this evidence highlights a key role for NEFL in the maintenance of axonal cytoskeleton and function.

### Multifaceted disruption of axonal homeostasis

#### AARS1

AARS1 is part of the aminoacyl-tRNA synthetase family and catalyzes the charging of alanine onto its cognate tRNA, which is delivered to the ribosome to participate in protein synthesis. The inclusion of a CE in the *AARS1* transcript has been observed in TDP-43 depleted neurons as well as in postmortem tissues from FTD-TDP and ALS-TDP patients (24, 51, 129, 158). The *AARS1* CE is inserted in-frame, predicted to be translated and to generate a novel protein containing a cryptic peptide in the catalytic domain of the protein. Interestingly, this cryptic peptide is able to trigger an immune response and T cell clonal amplification (35), highlighting the relevance of this event in ALS as an enrichment for clonally expanded CD4^+^ and CD8^+^ T cell populations has been observed in patients (167).

Importantly, dominant mutations in the *AARS1* gene cause Charcot-Marie-Tooth type 2N (CMT2N), a peripheral sensorimotor neuropathy. The mechanism through which *AARS1* mutations contribute to CMT2N is unclear, but genetic and functional data point to a GOF. Disease-linked mutants abnormally interact with Neuropilin-1, likely sequestering it and impairing its neurotrophic signaling pathway (140), and increasing evidence supports a role for aminoacyl-tRNA synthetases in the pathogenesis of neurological diseases, often leading to motor and sensory phenotypes (28). For example, CMT-causing GARS mutants aberrantly interact with TrkB (134), Neuropilin-1 (66) and HDAC6 (112), interfering with neurotrophic signaling and axonal transport respectively. Mutant GARS also abnormally binds tRNA^Gly^, causing ribosomal stalling and activating the integrated stress response pathway, which contributes to the peripheral neuropathy symptoms (135, 176). Recently, several studies have reported a new non-canonical function for AARS1 in protein lactylation in cancer, with consequences on gene expression (55). An intriguing possibility is that the presence of the cryptic peptide might affect AARS1 function and, similarly to previously reported CMT2-linked *AARS1* mutations, contribute to ALS pathogenesis through a toxic GOF or dominant-negative mechanism.

#### ELK1

TDP-43 LOF leads to an extension of *ELK1* 3’UTR, affecting transcript stability and ultimately leading to an increase in protein expression (25). ELK1 is a transcription factor involved in the regulation of gene expression, but mounting evidence in neurons points towards a cytoplasmic role as well (14). ELK1 was reported to localize to mitochondria, where it accumulates upon pro-apoptotic stimuli and its overexpression reduces cell viability through the opening of the mitochondrial permeability transition pore complex. Interestingly, local expression of ELK1 in dendrites is toxic in primary rat neurons (14). Recently, increased levels of ELK1 were detected in brain tissues of AD patients and model mice, and it was shown to affect the degradation of AD-related protein presenilin 1 leading to an increase in amyloid-β production (168). Therefore, it is conceivable that the increase in ELK1 levels associated with TDP-43 LOF might contribute to neuronal degeneration through similar pathways. An intriguing possibility is that the “cryptic” 3’UTR might also alter *ELK1* mRNA localization, leading to dendritic- and axonal-specific consequences.

#### AGRN

An in-frame CE has also been identified in the *Agrin* (*AGRN*) transcript, leading to the inclusion of a cryptic peptide in the protein (69, 129). Agrin has a key role in NMJ development, as it regulates the clustering of acetylcholine receptors, and is required for proper axonal innervation and NMJ formation in mice (111). *Agrn* mRNA is locally translated at NMJs in mice and abolishing its axonal localization disrupts NMJs integrity affecting synaptic transmission and motor ability (148). Interestingly, in mice lacking the RBP Nova, restoring the levels of the neuron-specific isoform *Z+ Agrin* was shown to be sufficient to rescue acetylcholine receptors clustering, although not motor function (126). *Agrn* mis-splicing and reduced levels of Z+ Agrin are also found in SMA mouse models (81, 173) and mutations in *AGRN* have been associated with congenital myasthenic syndrome, a neuromuscular disorder (71). This evidence highlights the importance of Agrin for motor neuron functionality. Although the inclusion of the cryptic peptide in Agrin does not alter its levels (129), it is possible that it might affect the functionality of the protein or its secretion, which has been shown to be important for signaling and synapse formation (71, 111). Further work on the characterization of the impact of CE-inclusion in *Agrin* will be relevant to elucidate its contribution to axonal and NMJ defects in TDP-43 proteinopathies.

Taken together, all these events affect genes involved in pathways essential for axonal maintenance such as proteostasis and translation (AARS1), neurotrophic signaling (AARS1), NMJ maintenance (AGRN), mitochondrial trafficking and function (ELK1), cytoskeleton regulation (STMN2 and NEFL). In this light, it is unsurprising that significant defects in axonal transport, NMJs, and axonal regeneration have been reported in ALS.

## The impact of TDP-43 loss on neuronal excitability and transmission

A large body of evidence from electromyography, transcranial magnetic stimulation and nerve conduction studies highlights synaptic dysfunction as an early event in ALS-FTD pathogenesis, with neuronal excitability changes detected at the pre-symptomatic stage (57) and correlating to disease progression and survival (78, 130). Riluzole is one of a handful of FDA-approved drugs for sporadic ALS based on its ability to reduce neuronal excitability, among other mechanisms of action (17). Both the nuclear and cytoplasmic pools of TDP-43 regulate synaptic transmission. TDP-43 RNP granules have been shown to localize to the pre- and post-synaptic terminals to regulate the transport and cue-dependent translation of synaptic proteins (16, 121), and TDP-43 regulates synaptic transmission through modulating the splicing of a subset of transcripts that encode synaptic proteins (23, 76, 99, 122).

### UNC13A

UNC13 are a family of proteins that are evolutionary conserved amongst primates (99). They coordinate multiple aspects of synaptic transmission (42). UNC13A is responsible for competent vesicular release in the majority of glutamatergic synapses (8, 152). Homozygous null *Unc13a* mice die shortly after birth due to poor respiration and feeding (8). Both LOF and GOF mutations in *UNC13A* cause a spectrum of neurodevelopmental disorders characterized by alterations in synaptic transmission, highlighting its crucial role in this process (7). Several genome-wide association studies (GWAS) have shown that single nucleotide polymorphisms (SNPs) in *UNC13A* are associated with increased sporadic ALS and FTD susceptibility and poor survival (159). The mechanisms underlying these associations remained unclear until research from our group along with Ward, Petrucelli and Gitler labs uncovered that TDP-43 depletion leads to the inclusion of a CE between exons 20 and 21, significantly reducing UNC13A protein level (23, 99). The *UNC13A* CE was exclusively detected in postmortem ALS-FTD tissue (23, 99). Two SNPs in *UNC13A*: *rs12973192* (C > G) and *rs12608932* (A > C), are located within the intron containing the CE, with the former located within the CE itself and the latter localizing 534 bp downstream of the CE donor splice site. While the location of the SNPs suggested they could directly impact *UNC13A* splicing, they were found to be insufficient to induce CE inclusion under physiological TDP-43 levels. Instead, the SNPs potentiate cryptic splicing by altering TDP-43 binding (23). The genetic correlation between the cryptic load and disease progression highlights UNC13A as a critical effector of TDP-43 neuropathogenesis. Indeed, restoring UNC13A protein level through an antisense oligonucleotide (ASO) targeting the CE or through CRISPR/Cas9-mediated deletion of *UNC13A* CE rescued the pre-synaptic deficits induced by TDP-43 depletion in iPSC-derived glutamatergic neurons (80). Thus, restoring *UNC13A* splicing is a promising therapeutic strategy that could expand the benefits of genomic medicine from the current ~2% achieved with Tofersen, an ASO for *SOD1-*ALS (110), to 97% of ALS-FTD cases, as well as other TDP-43 proteinopathies.

### SYT7

The synaptic transmission process is subject to short- and long-term strength changes, a process referred to as synaptic plasticity. Synaptic plasticity is the mechanism underlying experience-based remodeling of the neural circuitry, with these experiences being a learning event, a stressful experience or exposure to psychoactive compounds. Synaptotagmins (SYT) are a family of proteins that regulate calcium-dependent fusion events (161). SYT7 supports the docking and the replenishment of synaptic vesicles in hippocampal neurons (96, 154, 163). Depletion of TDP-43 introduces an out-of-frame CE between canonical *SYT7* exons 3 and 4 that destabilizes the transcript and accordingly diminishes the protein level (99, 129). Cryptic *SYT7* has been detected in the frontal cortex of ALS-FTD brains as well as hippocampus and amygdala of AD TDP-43 cases (48, 99). These findings suggest that efforts directed towards correcting TDP-43 mis-splicing events in ALS-FTD could extend to TDP-43 AD cases as well.

### KCNQ2

KCNQ2 is a member of the Kv7 family of voltage gated potassium (K^+^) channels. Together with KCNQ3, its expression is mainly neuronal and they form heteromeric channels producing an outward K^+^ current. These channels regulate M-currents, which are involved in the maintenance of resting membrane potential in unmyelinated portions of the axon, such as the axonal initial segment, the nodes of Ranvier and axonal terminals. Loss of the M-current lowers the threshold to trigger an action potential and reduces the length of the refractory period, contributing to neuronal hyperexcitability (63, 72). Therefore, it is not surprising that mutations in *KCNQ2* have been reported to cause epilepsy (113). Loss of TDP-43 leads to an exon skipping event in *KCNQ2* mRNA that does not result in a frameshift but instead leads to the production of a variant of KCNQ2 protein lacking the portion encoded by exon 5 (KCNQ2^ΔE5^) (129). This segment spans part of KCNQ2 transmembrane domain 5, extracellular components and a region of the pore-forming domain, which is crucial for K_+_ conductance. KCNQ2^ΔE5^ accumulates in the endoplasmic reticulum in neurons, leading to a dominant-negative effect (76). Remarkably, the resulting reduction of the M-current and associated hyperexcitability in TDP-43-depleted neurons can be rescued by correcting *KCNQ2* aberrant splicing through ASOs (76). Therefore, altered formation of Kv7 channels is likely to contribute to the hyperexcitability phenotypes observed in ALS, highlighting another pathway through which TDP-43-dependent mRNA processing ultimately influences synaptic transmission.

## Future directions: Harnessing cryptic exons for therapeutic benefits

Despite the complex interplay of genetic and environmental factors in ALS-FTD pathogenesis, the nuclear clearance of TDP-43 remains a unifying hallmark for ~97% and ~45% of the ALS and FTD cases respectively (106). Consequently, the discovery of cryptic splicing events among genes involved in neuronal function and survival as a pathomechanism for ALS-FTD paved the way for development of biomarkers that enable early disease diagnosis as well as therapeutic strategies that exploit splice-modifying methods to restore the correct splicing of CE-containing targets and their normal function (23, 30, 99, 147). While in some cases restoring functional protein levels can be readily achieved through an AAV (adeno-associated viral vector) that delivers the protein of interest to rescue the disease phenotype (109), this approach is not favorable for clinical purposes due to the absence of a braking system that prevents chronic overexpression and accordingly gain of toxic functions (150). For instance, long-term overexpression of survival motor neuron (SMN) in mice results in late-onset motor dysfunction reminiscent of the SMA phenotype (150). AAV-based strategies are further compounded by the limited packaging capacity of the vector and accordingly the size of the protein to be rescued - UNC13A for instance supersedes the typical capacity (103). Moreover, delivering the protein only to the affected neurons rather than the entire neuronal population presents a major challenge.

### Splice-switching ASOs and U7 snRNPs

One potential therapeutic strategy is the use of ASOs, single-stranded chemically modified oligonucleotides usually between 10 and 50 bp in length (21), that can be designed to bind sequences of interest with high specificity, triggering either the degradation of the targeted transcript through RNase H-dependent cleavage or altering pre-mRNA splicing (21). In the latter scenario, ASOs are targeted to splicing sites of interest and sterically block the access to the spliceosome, preventing its function (21). Importantly, several modifications have been optimized to increase ASOs stability and achieve efficient splicing modulation (21, 47). Nusinersen, an ASO that influences the splicing of exon 7 into *SMN2* mRNA, represents a milestone in the SMA field (151). Beyond extending the survival of SMA patients, nusinersen provided a proof of concept for the utility of ASOs as a forefront of therapeutic strategies in a spectrum of neurodegenerative disease (151). In the context of ALS, Tofersen is an FDA approved ASO utilized in rescuing toxic gain of functions observed in patients harboring *SOD1* mutations (110). While ASOs targeting *FUS* and *SOD1* have entered clinical trials, only 3% of all ALS cases are caused by mutations in these genes, limiting the number of patients benefiting from these treatments and highlighting an urgent need to identify targets that would extend the therapeutic benefit to the remaining 97% of cases displaying TDP-43 proteinopathy. To achieve this, a possible approach would be to target ASOs to CE sites, preventing their inclusion even in the absence of nuclear TDP-43 and potentially rescuing downstream phenotypes. Considering that ASOs correct individual splicing events, there is a need to identify those that significantly impact neuronal function and accordingly hold promise in improving the symptoms and/or halting disease progression. ASOs targeting *UNC13A, STMN2, ATG4B* and *KCNQ2* have been shown to rescue cryptic splicing in *in-vitro* models (12, 76, 80, 147). A clinical study evaluating the safety and efficacy of QRL-201, an ASO aiming to restore STMN2 levels in ALS patients, is currently underway (https://clinicaltrials.gov/).

However, ASOs have limitations. ASOs are typically administered intrathecally since they are unable to cross the blood brain barrier. Secondly, they require repeated dosing for continued response (21, 117, 139). While the long-term side effects of ASOs administration have not been clinically evaluated yet, the data from the ongoing clinical trials will be key in understanding the effects of cumulative dosages of ASOs, helping improve their suitability as ALS therapeutics.

ASOs have so far mostly been used to target single pre-mRNAs. Notably, although correcting *UNC13A* missplicing rescues presynaptic deficits (80), it does not normalize postsynaptic responses that are likely dependent on dendritically-localized TDP-43 RNP granules. The proper localization and function of these granules are probably further compromised by the cytoskeletal abnormalities and axonal accumulations discussed above. Importantly, while restoring UNC13A alone may enhance synaptic output, correcting the concurrent deficit in KCNQ2 that renders the neurons hyperexcitable may be important to restore normal function. These considerations highlight the need to evaluate not only single-target efficacy but also broader network-level outcomes to ensure safety and functional benefit. Indeed, it is important to consider that since the consequences of TDP-43 nuclear LOF are likely due to cryptic splicing events affecting several transcripts, strategies enabling the correction of multiple CE inclusions would offer a significant therapeutic advantage.

Another exciting therapeutic avenue is offered by a splice-modifying approach exploiting uridine-rich small nuclear ribonucleoproteins (U snRNPs), which are known for their role in pre-mRNA splicing. While most of them make up the spliceosome, the U7 snRNP specifically mediates the 3’ processing of replication-dependent histone pre-mRNAs. At its 5’ end, the U7 snRNA comprises an antisense small nuclear RNA (snRNA) which is complementary to 3’ end of the histone pre-mRNA while its 3’ sequence recruits 5 Sm proteins and 2 Sm-like proteins (Lsm10 and Lsm11), forming a heptamer ring. While the former are common to also the other snRNPs and form the spliceosome, Lsm10 and Lsm11 confer specificity for histone pre-mRNA processing and are necessary for this function. The U7 snRNP ability to bind RNA can be harnessed as a tool for splicing modulation. Indeed, replacing the consensus sequence for Sm-like proteins at the 3’ end of U7 snRNA with one for Sm proteins impairs the ability of the U7 RNP to target histone mRNA, as well as increasing its nuclear localization which is beneficial to achieve splicing regulation. Moreover, the 5’ region of the snRNA can be made complementary to any sequence in the transcriptome, targeting the U7 snRNP to any site of interest, where it can act as a modulator of splicing through steric hindrance. Remarkably, the addition of a tail to U7 snRNA to include binding sites for hnRNPA1 enhances exon skipping (53).

U7 snRNPs have already been used to modify the splicing of genes involved in diseases, such as β-globin for β-Thalassemia, *SMN2* for SMA and dystrophin for Duchenne muscular dystrophy (53). U7 snRNPs can also be engineered to alter splicing in order to achieve the specific degradation of mRNAs carrying mutations causing toxic gain of function, as reported for the ALS-causing *SOD1*^G93A^ mutant (15). Moreover, recent work shows that therapeutic U7 snRNAs can rescue *UNC13A* and *STMN2* CE inclusion, as well as associated cellular phenotypes, in TDP-43 depleted neurons and in a *STMN2* humanized mouse model (61, 107). Remarkably, expression of *STMN2*- and *UNC13A*-targeting snRNAs in the same vector can simultaneously correct cryptic splicing of both transcripts (61, 107).

Compared to ASOs, U7 snRNPs have several advantages. They efficiently accumulate in the nucleus, are amenable for gene therapy with a single dose being sufficient for life-long effect. Because of their small size, multiple U7 snRNAs can be packaged into a viral vector, allowing efficient delivery and targeting multiple cryptic events.

### CRISPR-associated nucleases

CRISPR-associated nucleases targeting either DNA or RNA can also be harnessed as gene therapy tools to correct cryptic splicing. CasRx is a ribonuclease and its deactivated version, dCasRx, enables targeting of specific RNA sequences and, either unconjugated or fused to a splicing regulator, it has been used to modulate exon inclusion or exclusion in the transcript of interest (115). The prime editing approach can be exploited to directly edit cryptic splicing sites in the genome, exploiting Cas9-based gene editing and avoiding highly mutagenic double-strand breaks (4, 39, 158).

All the approaches reported above constitute attractive options for the correction of cryptic splicing in ALS. However, it is important to note that the number of cryptic events to be targeted to halt disease progression is currently unclear, limiting the progression of these strategies into therapeutics.

### Rescuing TDP-43 splicing function more broadly

Restoring nuclear TDP-43 function through gene therapy represents a feasible approach to rescue the broad RNA processing defects resulting from its loss. However, it is important to consider that this strategy might not be achievable through expression of TDP-43 itself, as the already existing TDP-43 aggregates would likely sequester the exogenously delivered TDP-43 preventing it from performing its nuclear functions. Ling et al. tested whether the expression of a chimeric protein containing the TDP-43 RNA binding regions, allowing it to recapitulate TDP-43’s binding patterns, and a C-terminal domain derived from another RBP, allowing it to escape the TDP-43 aggregation sink, could rescue TDP-43 LOF. Expression of this chimera, where the aggregation-prone C-terminus of TDP-43 was replaced with the splicing repressor domain Raver1, rescued CE inclusion in TDP-43 depleted cells (94).

### Taking advantage of CEs: splice gated therapeutics

A number of the therapeutic approaches described above rely on bacterial or novel chimeric proteins, and their widespread expression could elicit an immune response or cause overexpression toxicity, thus limiting our ability to progress these as therapeutics. Indeed, while only a subpopulation of neurons display signs of pathology in ALS patients (158), gene therapy vectors are delivered to the majority of CNS cell populations rather than diseased neurons, potentially causing significant side effects through damage to healthy cells. This lack of precision is a major limitation for therapeutic approaches.

To overcome these limitations, recent work from our group has leveraged the specificity of cryptic splicing events resulting from the nuclear clearance of TDP-43 along with splice prediction tools to design vectors with synthetic cryptic splice sites that allow transgene expression only in cells exhibiting TDP-43 LOF, namely ‘TDP-REG’, thus considerably limiting potential side effects (158). In the TDP-REG system, an intron with a CE containing part of the coding sequence is inserted within the therapeutic gene of interest. The removal of the CE in healthy cells prevents translation of the therapeutic transgene and triggers the degradation of the RNA via NMD. Conversely, the loss of TDP-43 function in diseased cells promotes the inclusion of the CE, leading to the production of a functional therapeutic protein (158). Building on previous work (93, 94), TDP-REG has been coupled to the expression of chimeric TDP-43 proteins and has been shown to rescue multiple splicing events by driving the expression of the TDP-43/Raver1 chimera specifically in TDP-43 deficient cells (158). Notably, this system is compatible with AAV packaging, an already established delivery approach for gene therapies. The TDP-REG system can also be applied to prime editing approaches to target CE splice sites (158) or to drive the expression of any other protein coded therapy such as disaggregases, chaperones or intrabodies. By limiting the expression of these transgenes to the affected cells, toxicity can be minimized, thereby broadening the range of payloads that can be safely delivered through gene therapy in ALS and other TDP-43 proteinopathies.

## Perspective

While both TDP-43 LOF and GOF have been reported to play a role in ALS, the former has been shown to precede aggregate formation and to independently recapitulate disease phenotypes, suggesting that it is not merely a downstream consequence but rather a driver of a vicious pathogenic cycle that leads to neuronal degeneration. Although the upstream molecular event(s) that culminates in mislocalization remain incompletely understood, the identification of the downstream consequences sheds light on the pathways driving neurodegeneration in TDP-43 proteinopathies and offers unprecedented opportunities for the development of targeted therapeutics that would benefit the vast majority of ALS patients.

Splice-modulating approaches, such as ASOs and U7-based therapeutics, are particularly attractive considering their successful applications in other neuromuscular diseases. However, since TDP-43 regulates the processing of thousands of transcripts, it is foreseeable that correcting multiple splicing events would be necessary to achieve tangible clinical benefits.

The recent development of the TDP-REG system demonstrated how cryptic splicing can be leveraged to achieve expression of therapeutics specifically in cells with TDP-43 nuclear clearance. While the TDP-REG system can be applied to regulate the expression of virtually any construct, exploiting it to express less aggregation-prone TDP-43 variants offers the exciting possibility to fix all the downstream consequences of TDP-43 loss specifically in diseased cells. While this is an attractive scenario, the question of whether this would be sufficient to counteract the effects of TDP-43 GOF remains. In the event where TDP-43 nuclear clearance is indeed the trigger of the positive feedback loop sustaining TDP-43 aggregation, would breaking this circle by restoring TDP-43 nuclear levels in a controlled manner reinstate the lost balance, restraining further aggregation? Would this be sufficient to restore healthy gene expression patterns, suitable to sustain axonal and synaptic demands, in already challenged neurons? And if so, is there a critical period of time before which the therapy needs to be delivered to be effective?
